# Bioactivity-guided fractionation of the triglyceride-lowering component and *in vivo *and *in vitro *evaluation of hypolipidemic effects of *Calyx seu Fructus Physalis*

**DOI:** 10.1186/1476-511X-11-38

**Published:** 2012-03-14

**Authors:** Yihui Yang, Xianmei Piao, Mingyu Zhang, Xiaodan Wang, Bing Xu, Jiuxin Zhu, Zhiwei Fang, Yunlong Hou, Yanjie Lu, Baofeng Yang

**Affiliations:** 1 Department of Pharmacology (the State-Province Key Laboratories of Biomedicine- Pharmaceutics of China, Key Laboratory of Cardiovascular Research, Ministry of Education), Harbin Medical University, Harbin, Heilongjiang 150081, P. R. China

**Keywords:** Calyx seu Fructus Physalis, Hyperlipidemia, Triglyceride, Primary hepatocytes, HepG2 cells

## Abstract

**Background:**

In folklore, some people take the decoction of *Calyx seu Fructus Physalis *(CSFP) for lowering blood lipids. The present study is designed to evaluate the lipid-lowering activities of CSFP, and search for its pharmacodynamical material.

**Methods:**

CSFP was extracted by water and 75% ethanol, respectively. The extracts of CSFP for reducing serum lipid levels were evaluated on mouse model of hyperlipidemia. The optimized extract was subjected to the bioactivity-guided fractionation in which the liquid-liquid extraction, collumn chromatography, the *in vivo *and *in vitro *models of hyperlipidemia were utilized. The structure of active component was determined by ^13 ^C-NMR and ^1^H-NMR.

**Results:**

The 75% ethanol extract of CSFP decreased the serum total cholesterol (TC) and triglyceride (TG) levels in mouse model of hyperlipidemia. Followed a separation process for the 75% ethanol extract of CSFP, the fraction B was proved to be an active fraction for lowering lipid *in vivo *and *in vitro *experiments, which could significantly decrease the serum TC and TG levels in mouse model of hyperlipidemia, and remarkably decrease the increase of TG in primary mouse hepatocytes induced by high glucose and the increase of TG in HepG2 cells induced by oleic acid. The fraction B2, isolated from B on bioactivity-guided fractionation, could significantly decrease TG level in HepG2 cells. One compound with the highest content in B2 was isolated and determined as luteolin-7-O-beta-D-glucopyranoside by NMR spectra. It could significantly reduce the TG level in HepG2 cells, and inhibited the accumulation of lipids by oil red O stain.

**Conclusion:**

Our results demonstrated that the 75% ethanol extract of CSFP could improve *in vitro *and *in vivo *lipid accumulation. Luteolin-7-O-beta-D-glucopyranoside might be a leading pharmacodynamical material of CSFP for lowering lipids.

## Background

Hyperlipidemia, also known as hyperlipoproteinemia or high cholesterol, is caused by abnormal lipid and lipoprotein metabolism, which is a disorder characterized by abnormally high concentration of lipids (fats) in the blood that are correlated with the development of atherosclerosis, the underlying cause of coronary heart disease (CHD) and stroke [1]. Some evidence indicates that lipid-lowering treatment can reduce progression of coronary atherosclerosis [2]. Many medicinal plants have been found to be useful to successfully manage hyperlipidemia [3,4]. *Calyx seu Fructus Physalis *(CSFP), a commonly used Chinese herb, is the dried persistent calyx or the persistent calyx with fruits of *physalis alkekengi *L. var. franchetii (Mast.) Makino. The herbal preparations of CSFP are traditionally used to treat cough, sore throat, abscesses, and urinary problems. Currently, several pharmacological effects are reported such as antimicrobial [5], anti-inflammatory [6], antitumor [7], and so on, which might attribute to those identified compounds such as physalins, flavones, alkaloids, and so on [8]. The decoction of CSFP is believed to be useful as an antihyperlipidemic preparation. However, a survey of the literature revealed no hypolipidemic studies of this plant. The objectives of this study were to i) prepare and screen the active hypolipidemic extracts using animal models of hyperlipidemia, ii) isolate and demonstrate the active fraction(s) on bioactivity-guided separation, iii) identify and elucidate the underlying pharmacodynamical material.

## Results

### Lipid-lowering effects of the extracts of CSFP on mouse model of acute hyperlipidemia

**Figure 1:**
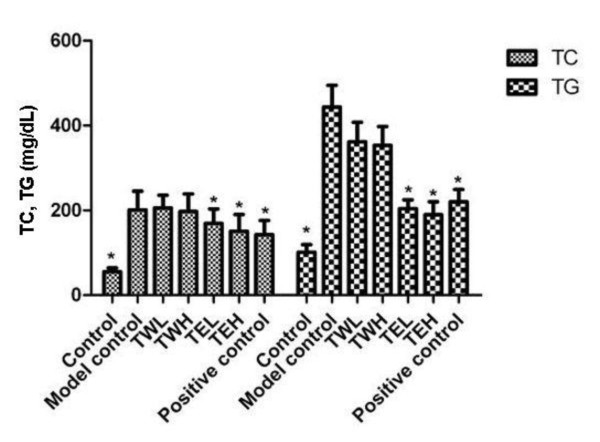
**Effects of the extracts of CSFP on TC and TG in mouse model of acute hyperlipidemia**. TWL: 1 g/kg/d; TWH: 2 g/kg/d; TEL: 1 g/kg/d; TEH: 2 g/kg/d; Positive control (Daming capsule): 0.5 g/kg/d. n = 8, * *p *< 0.05 compared with the model control group.

As shown in Figure 1, the increases in serum TC and TG levels induced in hyperlipidemic mice were prevented by prior administration of total 75% ethanol extract (TE), compared with the model control group. Total water extract (TW) had no statistically-significant effect compared to the model control.

### Lipid-lowering effects of E and B on mouse model of acute hyperlipidemia

**Figure 2:**
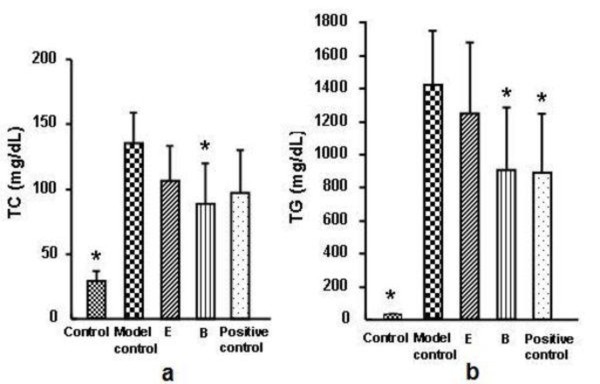
**Effects of E and B on TC and TG in mouse model of acute hyperlipidemia**. B: 0.54 g/kg/d; E: 0.24 g/kg/d; Positive control (Daming capsule): 0.5 g/kg/d. n = 6, * *p *< 0.05 compared with the model control group.

As shown in Figure 2, the increases in serum TC and TG levels induced in hyperlipidemic mice were prevented by prior administration of B, compared with the model control group.

### Lipid-lowering effects of fractions of CSFP on primary mouse hepatocytes and HepG2 cells

**Figure 3:**
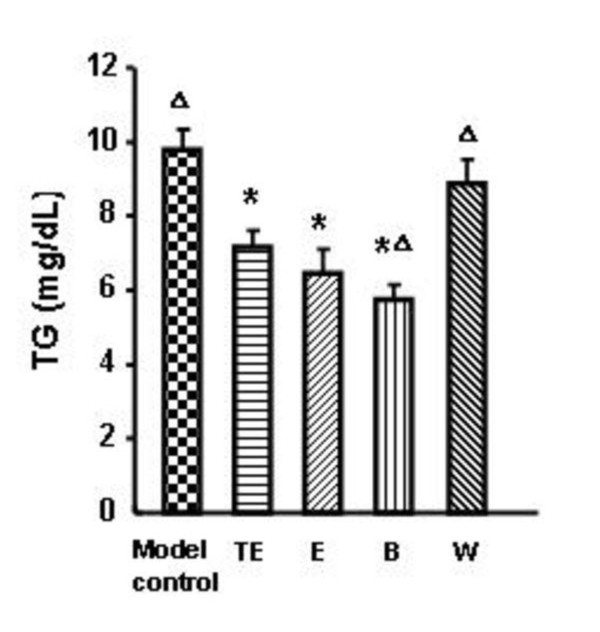
**Effects of TE, E, B, W on the TG content in primary mouse hepatocytes**. TE: 100 μg/mL; E: 100 μg/mL; B: 100 μg/mL; W: 100 μg/mL. n = 5, * *p *< 0.05 compared with the model control group, Δ *p *≤ 0.05 compared with TE group.

The cytotoxic activities of TE, E, B and W were detected by MTT. The result showed that they were not cytotoxic to primary mouse hepatocytes at the final concentration of 50 μg/mL (the data not shown). As shown in Figure 3, TE, E and B had statistically-significant effect to decrease the TG content in primary hepatocytes, compared with the model control group. Moreover, the effect of B was better than the effect of the cruder extract (TE).

**Figure 4:**
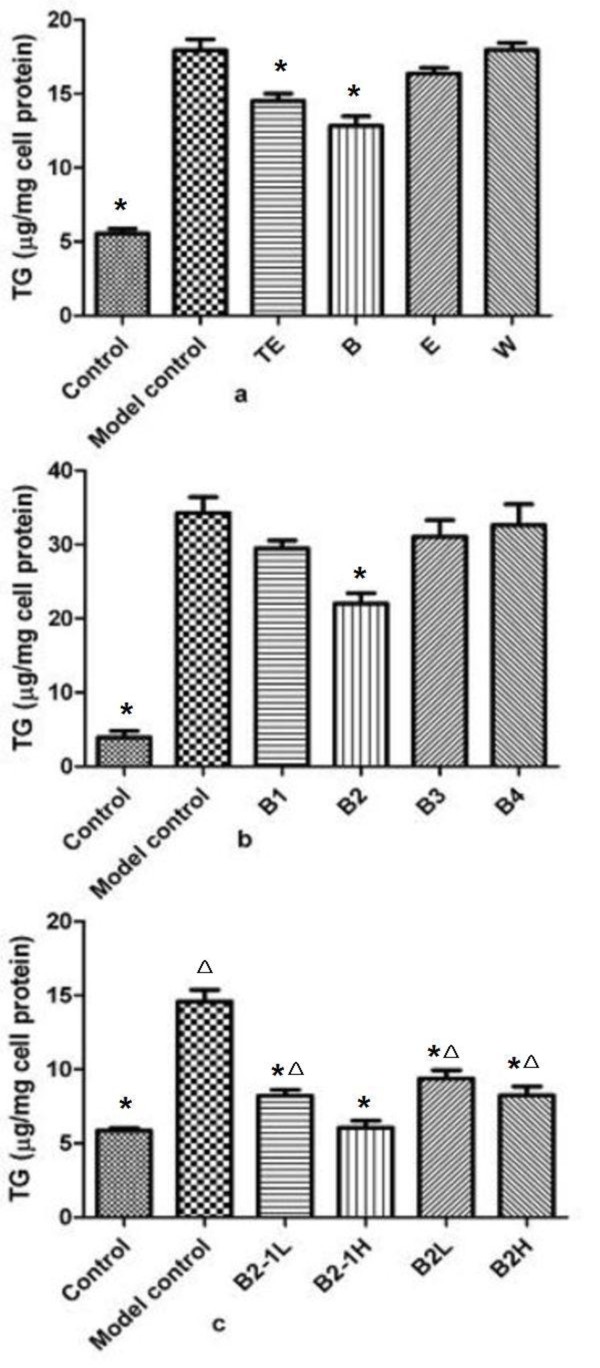
**Effects of the different fractions of CSFP on the TG content in HepG2 cells**. **a**. TE: 50 μg/mL; B: 50 μg/mL; E: 50 μg/mL; W: 50 μg/mL. **b**. B1: 50 μg/mL; B2: 50 μg/mL; B3: 50 μg/mL; B4: 50 μg/mL. **c**. B2-1L: 10 μg/mL; B2-1H: 50 μg/mL; B2L: 10 μg/mL; B2H: 50 μg/mL. n = 3, * *p *< 0.05 compared with the model control group, Δ *p *≤ 0.05 compared with the B2-1H group.

The MTT cytotoxicity test on HepG2 cells was used to determine the cytotoxic activities of TE, E, B, W, B1, B2, B3, B4 and B2-1. The result showed that they were not cytotoxic at the final concentration of 50 μg/mL (the data not shown). As shown in Figure 4a, TE and B had statistically-significant effect to decrease the TG content in HepG2 cells, compared with the model control group. The effects of E and B on mouse model of hyperlipidemia were consistent with that of E and B on primary mouse hepatocytes (Figure 3) and HepG2 cells (Figure 4a).

The Figure 4b showed that B2 had statistically-significant effect to decrease the TG content in HepG2 cells, compared with the model control group. A compound (B2-1) with the highest content in B2 was detected by HPLC and isolated by column chromatography. As shown in Figure 4c, B2-1 (50 μg/mL, 10 μg/mL) and B2 (50 μg/mL, 10 μg/mL) had statistically-significant effect to decrease the TG content in HepG2 cells, compared with the model control group. Moreover, the effect of B2-1 (50 μg/mL) was better than the effects of B2-1 (10 μg/mL) and B2 (50 μg/mL, 10 μg/mL).

### Oil red O staining of HepG2 cells treated with TE, B, B2 and B2-1

**Figure 5:**
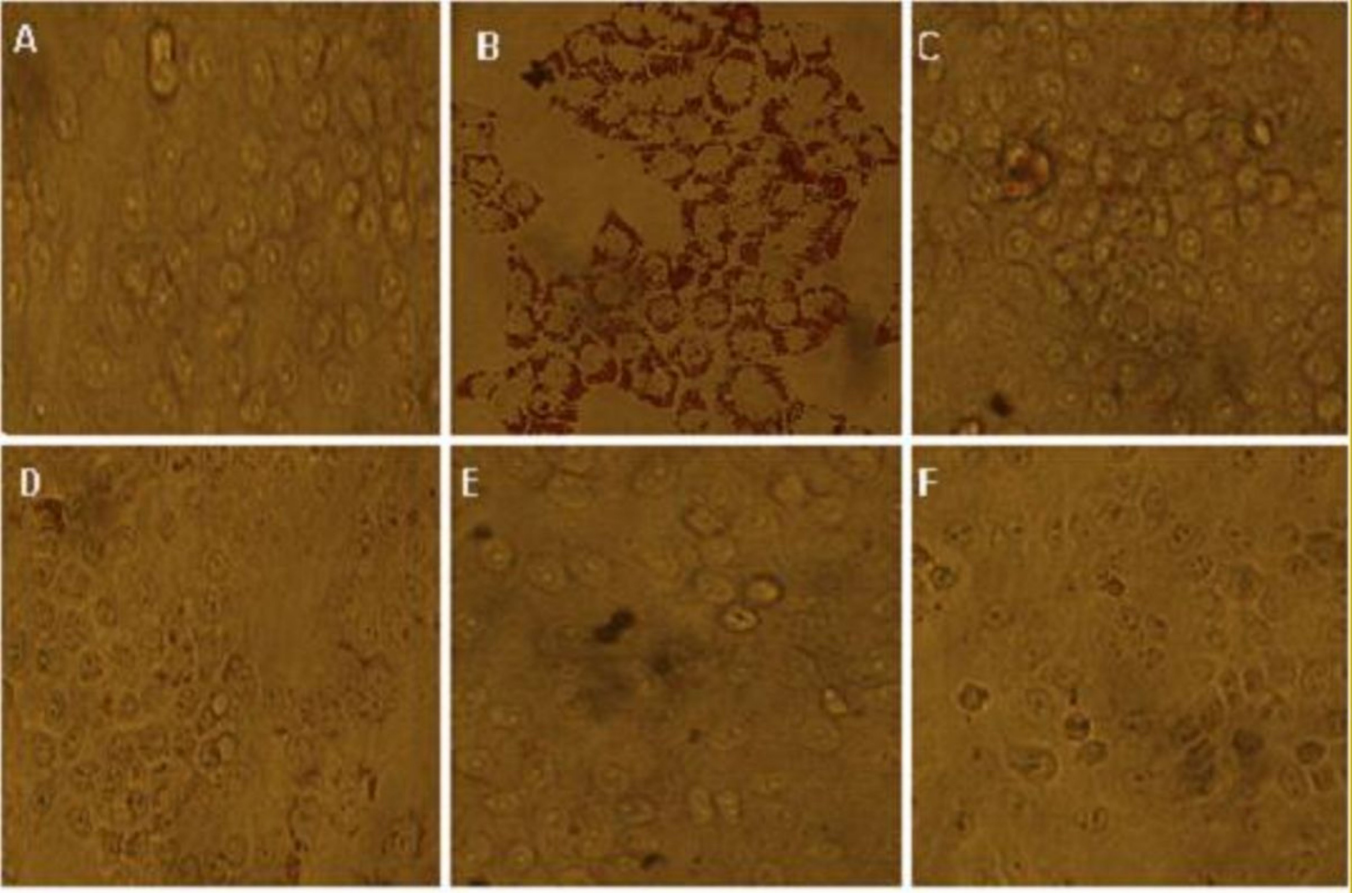
**Oil red O staining of HepG2 cells treated with TE, B, B2 and B2-1**. **A: **Control Group; **B: **Model Control Group; **C: **TE (50 μg/mL); **D: **B (50 μg/mL); **E: **B2 (50 μg/mL); **F: **B2-1 (50 μg/mL). Magnification ×400.

As shown in Figure 5, TE, B, B2 and B2-1 could remarkably improve the accumulation of lipids in HepG2 cells, compared with the model control group.

### Determination of B2-1

**Figure 6:**
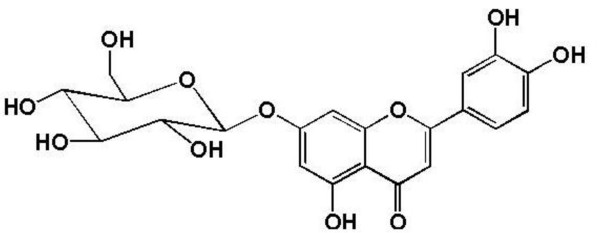
**Chemical structure of compound B2-1**.

B2-1: yellow powder; m.p. 257-259°C. It showed a dark spot on TLC under UV-light, but it turned into yellow fluorescence spot after it contacted with AlCl_3 _reagent. The results suggested that it might be a flavone. The result of molish reaction revealed that it might be a glycoside. Its structure (Figure 6) was determined by NMR spectra (300 MHz, DMSO-*d*_6_). All data of B2-1 were basically consistent with the data of luteolin-7-O-beta-D-glucopyranoside in the literature [9]. Thus, B2-1 was identified as luteolin-7-O-beta-D- glucopyranoside.

## Discussion

The traditional Chinese medicine (TCM) has been advocated for over 2,500 years by prescriptions, which is a unique medical system assisting the ancient Chinese in dealing with disease. Typically, TCM is well known for its formulae, which usually consist of several types of medicinal herbs. It is believed that, at least in some medicinal herbs, multiple components could hit multiple targets and exert synergistic therapeutic efficacies [10,11]. However, essential compounds from medicinal herbs have not been identified in most formulae, thus hampering the modernization of TCM. Much attention has been paid in the research on extracting active fractions or compounds from medicinal herbs to elucidate pharmacodynamical material bases during the past decades.

In folk medicine, the decoction of *Calyx seu Fructus Physalis *(CSFP) can be used to lower blood lipids. Although several kinds of compounds, such as physalins, flavones, and alkaloids, have been identified in this medicinal herb, there is no such study on evaluating the lipid-lowering pharmacotheraputic action, or elucidating the pharmacodynamical material bases. The present study was undertaken to investigate the lipid-lowering effect of CSFP, and search for the lipid-lowering fraction and compound. In order to evaluate the ethanolic and aqueous extracts of CSFP for antihyperlipidemic activity, the mouse model of acute hyperlipidemia was set up by intraperitoneal injection of egg yolk. The results showed the increases in serum TC and TG levels induced in hyperlipidemic mice were prevented by prior administration of the 75% ethanol extract (TE).

In order to elucidate the active fractions and components, a bioactivity-guided separation on the 75% ethanol extract was performed. Firstly, the 75% ethanol extract was divided into fraction E, fraction B and fraction W by liquid-solid extraction. The primary mouse hepatocytes induced by high glucose and HepG2 cells induced by oleic acid were employed as the *in vitro *screening models for lipid-lowering activity. The results showed that the lipid-lowering activity might attribute to the fraction B, which were confirmed by the *in vivo *experimental results of acute hyperlipidemic mice. The results demonstrated that the lipid-lowering response of HepG2 cells were consistent with primary mouse hepatocytes and acute hyperlipidemic mice. HepG2 cells were used in subsequent experiments.

In order to isolate and identify its pharmacodynamical material bases, the fraction B was divided further into Part B1, Part B2, Part B3 and Part B4 by macroporous adsorbent resins column chromatography. The screening experiment for lipid-lowering fractions was reproduced in HepG2 cells induced by oleic acid. The results demonstrated that Part B2 had significant effect to decrease TG level in HepG2 cells. The maximal chromatographic peak was detected in 3D chromatogram of Part B2 by HPLC, and its peak area was up to 80% of total peak area at 350 nm. The main component (B2-1) was isolated from Part B2 by silica gel and polyamide column chromatography, and identified as luteolin-7-O-beta-D-glucopyranoside by ^1^H NMR and ^13^C NMR, which significantly decreased the TG level in HepG2 cells.

*Calyx seu Fructus Physalis *contains many kinds of flavones, such as luteolin, ombuine, luteolin-7-O-beta-D-glucopyranoside, luteolin-4'-O-beta-D-glucopyranoside, quercetin-3-O-beta-D-glucopyranoside, luteolin-7,4'-di-O-beta-D-glucopyranoside, and so on [12,13]. It is reported that many flavonoids have lipid-lowering effects [14,15]. Luteolin has been reported to reduce the accumulation of lipids in HepG2 cells [16]. Luteolin-7-glucoside may improve lipid profiles in rats [17]. All those informations support a hypothesis that the flavones in *Calyx seu Fructus Physalis *might be lipid-lowering pharmacodynamical material bases. In our experiments, luteolin-7-O-beta-D- glucopyranoside remarkably reduced the increase of TG in HepG2 cells induced by oleic acid. So it was demonstrated that luteolin-7-O-beta-D- glucopyranoside might be one of major lipid-lowering components of *Calyx seu Fructus Physalis*.

In addition, high intrahepatic levels of TG and cholesterol do not necessarily correlate with high serum levels of these lipids. But the body can reduce serum levels by increasing hepatic lipid uptake from the serum and/or decreasing secretion from the liver. There is an argument for the utility of the cell systems for screening lipid-lowering compound. So it is necessary to evaluate the active compound (luteolin-7-O-beta-D-glucopyranoside) in animal model in future, to verify the validity of the cell-based assays predicting *in vivo *effectiveness.

## Conclusion

Our study clearly demonstrated that 75% ethanol extract of *Calyx seu Fructus Physalis *inhibited *in vitro *and *in vivo *lipid accumulation. In addition, luteolin-7-O-beta-D-glucopyranoside might contribute to lipid-lowering activities. We suggest that 75% ethanol extract of *Calyx seu Fructus Physalis *has high therapeutic potential for prevention of hyperlipidemia. However, clinical trial in hyperlipidemic patients is necessary in future.

## Materials and methods

### Animals, cell line, chemicals and biochemicals

Kunming mice(KM) were provided by Laboratory Animal Department of Harbin Medical University. All animal procedures were approved by the Ethical Committee for Animal Experiments, Harbin Medical University, and confirmed with the Guide for the Care and Use of Laboratory Animals published by the US National Institutes of Health (NIH Publication No. 85-23, revised 1996). HepG2 cells (Human Hepatocellular Carcinoma) were purchased from Cell Resource Center of Shanghai Institute for Life Sciences, Chinese Academy of Sciences. Daming capsule, as positive hypolipidemic drug in animal experiments, was purchased from Harbin Yida pharmaceutical Co., Ltd, China. Daming capsule (a traditional Chinese formula) was composed with *Radix et Rhizoma Rhei, Semen Cassiae*, etc. and used to treat hyperlipoidemia in clinic [18]. The kits for TC and TG were purchased from Bio Sino Bio-technology and Science Inc., China. The oleic acid was purchased from Sigma. The cell lysis buffer and BCA Protein Assay Kit were purchased from Beyotime. The mammalian protease inhibitor was purchased from Shanghai Biocolor BioScience & Technolgy company.

### Plant material

*Calyx seu Fructus Physalis*, purchased from the Medicinal Material Company of Shiyitang in Harbin, China, was identified as the persistent calyx of *physalis alkekengi *L. var. franchetii (Mast.) Makino by Dr. Guoyu Li of Harbin Medical University. A voucher specimen of CSFP (Registration number: JDL-20090826) was deposited in the herbarium of Harbin Medical University.

### Extraction and isolation

CSFP (100 g) were extracted with water (3 × 1000 mL, 3 h each) at 80°C to give the total water extract (TW, 26 g, 26%). CSFP (1000 g) were extracted with EtOH-H_2_O 75:25 (3 × 10000 mL, 3 h each) at 80°C to give the total 75% ethanol extract (TE, 208 g, 20.8%). TE (165 g) was extracted by ethyl acetate (2 × 1000 mL, 2 h each), n-butanol (2 × 1000 mL, 2 h each) and water (2 × 1000 mL, 2 h each) at 80°C, respectively. The corresponding extracts were obtained, such as part E (20 g, 12%), part B (45 g, 27%) and part W (100 g, 61%). Part B (18 g) was subjected to macroporous resin column (D101, 6 × 50 cm, flow rate 4 mL/min, Chemical Plant of Nankai University) using a step gradient of EtOH-H_2_O 0:100 (4 L), 30:70 (4 L), 60:40 (4 L) and 97:3 (4 L). Fractions of 4 L were collected and they were B1 (3.2 g), B2 (5 g), B3 (4 g) and B4 (2.3 g), respectively. B2 was analyzed by HPLC (Agilent 1200 Infinity Series, USA) on a C18 column (5 μm, 4.6 × 250 mm, Nacalai Tesque, Japan) eluted with MeOH-H_2_O 50:50 at 1 mL/min. A compound (B2-1) with the highest content in B2 was detected in the 3D chromatogram of HPLC and the t_R _of B2-1 was 7.4 min. The compound B2-1(35 mg) was isolated from B2 (4 g) on polyamide column (48-75 μm, 3 × 30 cm, Chemical Plant of Shanghai) and silica gel column (38-75 μm, 2 × 25 cm, Qingdao Haiyang Chemical Co., Ltd). The chemical structure of B2-1 was determined by NMR spectra (Bruker AVANCE 300, Switzerland). The samples of cell experiments were dissolved in the ddH_2_O with 4% DMSO. The final concentration of DMSO was 0.1% in culture medium.

### Mouse model of hyperlipidemia

After the extracts were orally administrated to KM mice once per day for 7 days, the mice were intraperitoneally injected with 75% yolk emulsion (20 ml/kg) for making mouse model of acute hyperlipidemia except for the control group. Twenty hours later, the blood sample was drawn from abdominal aorta, centrifugated and collected supernatant. The contents of TC and TG in serum were determined by kits.

For evaluating the lipid-lowering effects of 75% ethanol extract of CSFP, fifty-six male KM mice (28-32 g) were randomly divided into seven groups: control group, model control group, TW (1 g/kg/d), TW (2 g/kg/d), TE (1 g/kg/d), TE (2 g/kg/d) and positive control drug group (0.5 g/kg/d, Daming capsule).

For evaluating the lipid-lowering effects of part E and part B, forty male KM mice (28-32 g) were randomly divided into five groups: control group, model control group, E group (2 g/kg/d × 12% = 0.24 g/kg/d), B group (2 g/kg/d × 27% = 0.54 g/kg/d) and positive control drug group (0.5 g/kg/d, Daming capsule). The dosages of E and B were correlated with their extraction rates.

### Preparation of primary mouse hepatocytes

Primary mouse hepatocytes were isolated by Seglen two steps perfusion method [19]. At first, the mouse was anaesthetized by pentobarbita. The peritoneal cavity was opened, and the portal vein was catheterized. The liver was perfused (5 mL/min, 37°C) with the perfusate (137 mM NaCl, 5.4 mM KCl, 0.34 mM Na_2_HPO_4_, 0.44 mM KH_2_PO_4_, 4.2 mM NaHCO_3_, 10 mM HEPES, 0.5 mM EGTA, 5.6 mM glucose, PH 7.2) for 2 min. The liver was perfused again with the perfusate (0.5 mg/mL collagenase, 137 mM NaCl, 5.4 mM KCl, 0.34 mM Na_2_HPO_4_, 0.44 mM KH_2_PO_4_, 4.2 mM NaHCO_3_, 10 mM HEPES, 0.5 mM CaCl_2_, 5.6 mM glucose, PH 7.5). After the digestion, the liver was put into culture medium, cut into pieces and filtrated. Filtrate was centrifugated (1 min, 700 rpm and 4°C), and the supernatant was removed. Culture medium was added into the centrifuge tube, and the cells were resuspended by repeated pipetting. The density of the cells was adjusted to be 2.5 × 10^5 ^cell/mL by supplementing culture medium. Finally, the cells were transfered into 96-well plate and cultured in high-glucose DMEM supplemented with 10% FBS and 1% penicillin-streptomycin under a humidified atmosphere of 5% CO_2 _at 37°C.

### High glucose induced up-regulation of TG on primary mouse hepatocytes

Our experiment had testified that the TG content of primary mouse hepatocytes in high-glucose culture medium was 2 ~ 3 times than that in low-glucose culture medium. The results showed that the high-glucose condition promoted hepatocytes to produce more TG. The primary mouse hepatocytes were cultured at high-glucose condition in 96-well plate, and then treated for 48 h with TE, E, B and W, respectively. The cells were washed with cold phosphate-buffered saline (PBS), resuspended in ddH_2_O and disrupted by ultrasonication. The TG content in cells was determined by kits.

### Oleic acid induced up-regulation of TG on HepG2 cells

The lipid composition of HepG2 cells was close to that of normal human liver cells. It was used as cell model for lipid-lowering research. The cells were cultured in high-glucose DMEM supplemented with 10% FBS and 1% penicillin-streptomycin under a humidified atmosphere of 5% CO_2_. After the cells adhered and grew in 6-well plate, the oleic acid with the final concentration of 500 μM was added into culture medium to stimulate cells to produce more TG as model cells [20]. The control group cells were cultured without oleic acid. The HepG2 cells were treated for 48 h with different fractions, respectively.

### Measurement of TG content in HepG2 cells

HepG2 cells in 6-well plate were washed with cold PBS, and then digested by trypsin. After the cells were centrifugated at 1000 rpm for 3 min, the supernatant was removed and the cells were washed with PBS. The cells were centrifugated again and the supernatant was removed, and then the cells were added into 400 μL of isopropanol at 4°C for 2 h. After the isopropanol solution was centrifugated (16000 rpm, 10 min, 4°C), the supernate was evaporated until dry. The residua were added into 20 μL of isopropanol, then to determine the TG content by kits [21]. The sediment of centrifugation at 16000 rpm was added into 40 μL of cell lysis buffer with 1% mammalian protease inhibitor, disrupted by ultrasonication and centrifugated (13500 rpm, 15 min, 4°C). The content of protein in supernate was determined by BCA Protein Assay Kit. The intracellular TG content was represented as μg/mg cell protein.

### Oil red O stain

For examination of fat accumulation in HepG2 cells, the cells were treated for 48 h with different fractions of CSFP. The cells were rinsed with cold PBS and fixed in 10% paraformaldehyde for 30 min. After the cells were washed with 60% isopropanol, the cells were stained for at least 1 hour in a freshly diluted Oil Red O solution (six parts Oil Red O stock solution and four parts H_2_O; Oil Red O Stock solution is 0.5% Oil Red O in isopropanol) [22]. After the stain was removed and the cells were washed with 60% isopropanol, the image of each group was photographed.

### Statistical analysis

All the data were expressed as mean ± standard deviation (SD). Data were statistically analyzed by one-way ANOVA, followed by Student-Newman-Keuls test to determine differences between groups. All statistical analyses were performed with SPSS 13.0 for Windows (SPSS Inc., Chicago, IL). Values of *p *< 0.05 were regarded as significant difference.

## Competing interests

The authors declare that they have no competing interests.

## Authors' contributions

YY and XP participated in experimental design, carried out experiments, and drafted the manuscript. MZ, XW and BX carried out experiments about isolation and identification of chemical components. JZ, ZF and YL carried out experiments about bioactivities. BY and YH conceived of the study, participated in experimental design, and drafted the manuscript. All authors read and approved the final manuscript.
